# Functionalization-Dependent Cytotoxicity of Silver Nanoparticles: A Comparative Study of Chlorhexidine and Metronidazole Conjugates

**DOI:** 10.3390/biom15060850

**Published:** 2025-06-10

**Authors:** Karol P. Steckiewicz, Monika Dmochowska, Elżbieta Megiel, Ewelina Barcińska, Iwona Inkielewicz-Stępniak

**Affiliations:** 1 Department of Pharmaceutical Pathophysiology, Faculty of Pharmacy, Medical University of Gdansk, 80-211 Gdansk, Poland; karol.steckiewicz@gumed.edu.pl (K.P.S.); mdmochowska@zoo.gda.pl (M.D.);; 2 Department of Anesthesiology and Intensive Therapy, Faculty of Medicine, Medical University of Gdansk, 80-214 Gdansk, Poland; 3 Faculty of Chemistry, University of Warsaw, 02-093 Warsaw, Poland

**Keywords:** silver nanoparticles, cell death, cytotoxicity, oxidative stress, functionalization-dependent cell death

## Abstract

This study examines the cytotoxicity of two silver nanoparticle formulations—AgNPs conjugated with chlorhexidine (AgNPs-CHL) and AgNPs conjugated with polyethylene glycol and metronidazole (AgNPs-PEG-MET)—as examples of the surface functionalization of silver nanoparticles with drugs via sulfur–silver bonds and nitrogen–silver interactions. We previously reported the synthesis of these NPs and their efficiency in periodontitis treatment. Here, we analyze the relationships between the cytotoxic mechanisms of AgNPs and their surface chemistry. UV–Vis spectroscopy, dynamic light scattering (DLS), and scanning electron microscopy (SEM) with energy-dispersive X-ray spectroscopy (EDX) were used for physicochemical studies of the conjugates in two environments: aqueous solutions and commonly used cell culture media. Cytotoxicity was assessed in human fetal osteoblasts (hFOB 1.19) and human gingival fibroblasts (HGF-1) through BrdU and LDH assays, ROS detection, cell cycle analysis, apoptosis assays, and protein expression studies. AgNPs-CHL showed aggregation and increased hydrodynamic diameters in the culture medium, while AgNPs-PEG-MET remained stable. Both exhibited concentration-dependent cytotoxicity: AgNPs-CHL at 0.4–10 μg/mL and AgNPs-PEG-MET at 0.75–10 μg/mL. AgNPs-CHL, in which silver surface functionalization was realized via nitrogen–silver interactions, induced significant ROS generation, LDH release, and necroptosis, marked by increased RIP1, RIP3, and MLKL proteins. In the case of AgNPs-PEG-MET, where sulfur–silver bonds combined the drug via a PEG linker, they triggered apoptosis, as evidenced by elevated caspase-2 levels and flow cytometry. These findings highlight that the type of surface functionalization of silver nanoparticles significantly influences their physicochemical behavior and biological effects. Understanding these mechanisms is crucial in designing safer, more effective nanoparticle-based therapies for periodontal and other inflammatory conditions.

## 1. Introduction

Nanoparticles (NPs) are increasingly being explored in biomedical applications due to their unique physicochemical properties, which differ markedly from those of their bulk counterparts. Metal-based NPs, in particular, have shown promise as antimicrobial, anti-inflammatory, and anticancer agents, and several studies have proposed their use as platforms for targeted drug delivery [1,2,3,4,5,6]. Silver nanoparticles (AgNPs), in particular, have received considerable attention in recent years for applications in wound healing, implant coatings, and dental materials [7,8,9]. Their high surface-to-volume ratio and ability to release Ag⁺ ions facilitate their biological activity.

Among metal NPs, silver nanoparticles (AgNPs) are especially well studied due to their broad-spectrum antimicrobial properties. However, their translation into clinical use is challenged by concerns over their cytotoxicity. The primary mechanism by which AgNPs exert toxicity involves the induction of oxidative stress via reactive oxygen species (ROS) generation. Additional pathways, including mitochondrial dysfunction, membrane damage, inflammatory cytokine release, and intracellular calcium dysregulation, have also been implicated [10,11,12,13]. These stress responses may lead to various modes of cell death, such as apoptosis, necrosis, autophagy, or necroptosis [12,13]. Moreover, AgNPs may induce genotoxic effects and DNA fragmentation [14,15].

Importantly, recent studies emphasize that the surface chemistry—including functionalization and capping agents—plays a critical role in modulating the biological behavior of AgNPs [16,17,18]. Functional groups on the AgNPs’ surfaces influence not only their stability and cellular uptake but also the type and extent of their cytotoxicity. Surface-bound ligands can alter protein corona formation, intracellular localization, and oxidative stress responses [19]. However, the mechanisms by which different functionalization strategies influence cell fate are not fully understood.

Recently, we reported that silver nanoparticles conjugated with chlorhexidine (AgNPs-CHL) and silver nanoparticles conjugated with polyethylene glycol (PEG) and metronidazole (AgNPs-PEG-MET) may serve as drug delivery platforms in periodontitis [20]. This approach is supported by recent research highlighting the therapeutic potential of AgNPs loaded with metronidazole or chlorhexidine in periodontal applications [21,22,23]. We reported the antibacterial and anti-inflammatory properties of AgNPs-CHL and AgNPs-PEG-MET. We determined that both AgNPs were active against both planktonic and biofilm forms of several bacterial and fungal strains that may be present in the oral cavity. We also determined that the developed AgNPs decreased the production of pro-inflammatory cytokines (IL-1β, IL-6, IL-8, and TNF-α) in lipopolysaccharide (LPS)- stimulated murine macrophage cells (RAW264.7). Again, AgNPs-CHL were more potent [20]. Although AgNPs-CHL and AgNPs-PEG-MET certainly had beneficial properties, they were not free of cytotoxicity. A complete understanding of the cytotoxicity of potential pharmaceuticals is crucial for their safe use. This work aimed to understand the cytotoxicity mechanisms of AgNPs-CHL and AgNPs-PEG-MET. Obtaining this knowledge will also enable the better tailoring of future synthesis processes for biomaterials to produce safer and more effective NPs.

## 2. Materials and Methods

### 2.1. Nanoparticles

**Scheme 1:**
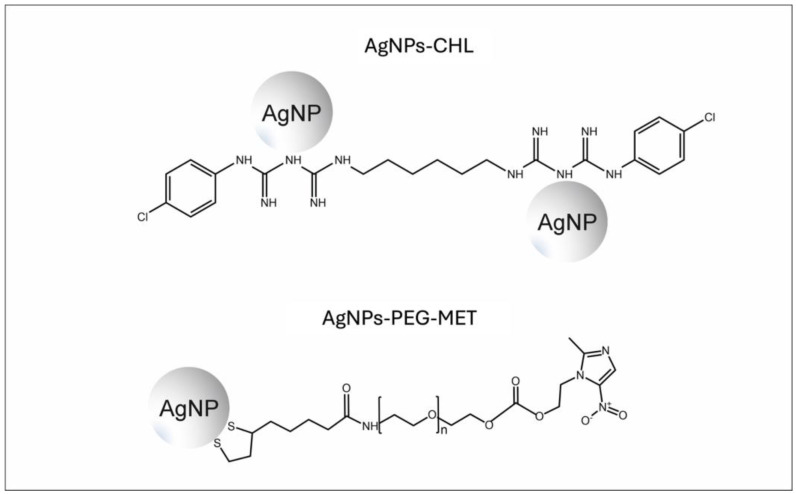
Structure of the studied conjugates of chlorhexidine or metronidazole with silver nanoparticles.

The previously described AgNPs-CHL and AgNPs-PEG-MET were used in this study [15]. Chlorhexidine is directly connected to the silver surface via a PEG linker with metronidazole. We hypothesized that there would be differences in toxicity for this reason, as the silver surface in AgNPs-CHL is more accessible and available than in AgNPs-PEG-MET. As in the previous case, the silver surface is tightly coated with a PEG polymer layer attached via stronger sulfur–silver bonds rather than the weak nitrogen–silver interactions seen in AgNPs-CHL (Scheme 1).

### 2.2. UV–Vis Studies of Nanoparticles’ Stability

The nanoparticles were dispersed in ultrapure water at a concentration of 500 ppm and supplemented with the medium used for cell culturing (200 µL per 1 mL of the nanoparticles’ solution). UV–Vis spectra were recorded using a UV–Vis spectrophotometer 1900i (Shimadzu Corporation, Kioto, Japan).

### 2.3. Dynamic Light Scattering (DLS) and ζ-Potential Studies

DLS measurements were performed using a Zetasizer Nano series apparatus (Malvern Pananylitical, Marven, UK) with backscattering detection at a constant 173° scattering angle, equipped with a He-Ne laser (4 mW) at 632.8 nm and a thermostated cell holder. The hydrodynamic diameters of the particles were measured at 25 °C. The size distribution by number was determined in ultrapure water, as well as the medium used for cell culturing. The surface’s zeta potential (ζ-potential) was determined for the same solutions as in the hydrodynamic diameter measurements, using Zetasizer folded capillary cells for electrophoresis.

The solutions for the analyses were prepared in the same way as for the UV–Vis stability studies (see Section 2.2).

### 2.4. Scanning Electron Microscopy (SEM)

Scanning electron microscopy with energy-dispersive X-ray spectroscopy (SEM-EDS) measurements were carried out on the nanoparticle solutions, both without and with the addition of a medium for cell culturing, using a scanning electron microscope (JEOL-JSM-5600 SEM-EDS, Jeol ltd, Akishima, Japan) equipped with an energy-dispersive X-ray spectrometer (Oxford—Link ISIS 300, Oxford Instruments, Abingdon, UK).

### 2.5. Cell Culture

Human fetal osteoblast (hFOB1.19) and human gingival fibroblast (HGF-1) cells were used in this study. hFOB1.19 cells were cultured in  a 1:1 mixture of Ham’s F12 Medium and Dulbecco’s Modified Eagle’s Medium supplemented with 2.5 mM L-glutamine, whereas HGF-1 cells were cultured in Dulbecco’s Modified Eagle’s Medium. Both media were supplemented with 10% fetal bovine serum (FBS) and 1% penicillin/streptomycin. Cell culture was performed according to the American Type Culture Collection’s recommendations (37 °C, in a humidified atmosphere of 5% CO_2_).

### 2.6. Treatments

hFOB1.19 and HGF-1 cells were exposed to either AgNPs-CHL or AgNPs-PEG-MET for 24 h. The specific concentrations employed in these experiments were established through prior investigations. Before each experiment, AgNPs were diluted in media without fetal bovine serum (FBS) and thoroughly mixed to ensure the uniform dispersion of AgNPs within the solution. Control samples were subjected to culture media lacking AgNPs and fetal bovine serum (FBS). Throughout the incubation period, the culture medium remained unchanged. In this study, we used concentrations that exerted beneficial antimicrobial and anti-inflammatory effects, as determined in our previous study [20].

### 2.7. BrdU Assay

We employed the BrdU proliferation ELISA kit from Roche to assess cell proliferation. Both hFOB1.19 and HGF-1 cells were used in this assay. These cells were seeded in a 96-well dish and then subjected to AgNP treatment within a concentration range of 0.2–10 μg/mL, as detailed in Section 2.6. Subsequently, we determined the antiproliferative effects of the AgNPs by measuring BrdU incorporation, following the manufacturer’s protocol. The data are presented as a percentage of the control group, where the proliferation rate of the control cells was set at 100%. Absorbance values were adjusted by subtracting the blank AgNP reading.

### 2.8. LDH Assay

We assessed cell viability using the lactate dehydrogenase (LDH) assay (Promega GmbH, Walldorf, Germany). To conduct this analysis, hFOB 1.19 and HGF-1 cells were seeded into a 96-well plate. These cells were exposed to AgNPs on the following day, as described above. We quantified the release of LDH into the surrounding medium, following the manufacturer’s instructions. Absorbance values were adjusted by subtracting the blank AgNP reading. The LDH data were then presented as a percentage of the total LDH released from the cells into the culture medium.

### 2.9. Detection of Reactive Oxygen Species

HGF-1 cells were seeded in 6-well plates. The following day, the existing medium was replaced, and the cells were treated with AgNPs, as described in Section 2.6. Following incubation, the culture media were removed and replaced with a fresh solution containing 10 μM of 2,7-dichlorofluorescein diacetate (DCF-DA). After a 30 min incubation period, the fluorescence emitted by oxidized DCF was quantified using flow cytometry, with excitation at a wavelength of 480 nm and emission at 525 nm. The data were expressed as a percentage relative to untreated cells, where the untreated cells were set at 100%. The analysis was performed using a BD FACS Calibur cytometer (Becton Dickinson, Franklin Lakes, NJ, USA) and the FlowJo v10 software (FlowJo LLC, Ashland, OR, USA).

### 2.10. Cell Cycle Analysis

HGF-1 cells were seeded in 6-well plates and subjected to the same treatment regimen outlined in Section 2.6. Following incubation, the cells were carefully washed, collected, and fixed using 70% ethanol at 4 °C. Subsequently, the fixed cells were centrifuged and resuspended in PBS containing RNAse A (50 μg/mL) and propidium iodide (50 μg/mL). After 30 min of incubation, the samples were analyzed using flow cytometry, a BD FACSCalibur cytometer (Becton Dickinson, Franklin Lakes, NJ, USA), and the FlowJo software (FlowJo LLC, Ashland, OR, USA). To ensure accuracy, FSC/SSC and FL2-A/FL2-W plots were utilized to exclude doublets and debris. The software was then employed to determine the number of cells in each cell cycle phase, with a minimum sample size of 15,000 cells.

### 2.11. Apoptosis Detection

We utilized an apoptosis assay kit (BD Pharmingen, San Diego, CA, USA), which employed Annexin V binding and propidium iodide (PI) uptake, to distinguish between apoptotic and necrotic cells. HGF-1 cells were seeded in a 6-well plate. The following day, these cells underwent treatment with AgNPs, as previously described. Subsequently, the cells were harvested, subjected to two washes with PBS, and then resuspended in a binding buffer. A mixture of 5 μL of Annexin V and 5 μL of propidium iodide was gently mixed into the cell suspension. After a 15 min incubation period at room temperature in the dark, the cells were diluted in the binding buffer and analyzed using a BD FACS Calibur cytometer and the FlowJo software. Plots generated from the gated cells illustrated the populations present, including viable (Annexin V−PI−), apoptotic (Annexin V+PI−), apoptotic/necroptotic (Annexin V+PI+), and dead (Annexin V−PI+) cells.

### 2.12. Western Blotting

We conducted Western blot analysis to assess the impact of AgNPs on protein expression, following a previously established and described method. In brief, HGF-1 cells were initially seeded into 100 mm Petri dishes. The medium was replaced upon reaching confluence, and the cells were treated with AgNPs as specified in Section 2.6. After a 24 h incubation period, the culture medium was aspirated, and the cells were washed, detached, and subsequently lysed. Protein concentrations were determined using the Bradford method, and samples were prepared for electrophoresis. Electrophoresis was performed to separate proteins and transfer them to nitrocellulose membranes (Protran, Schleicher, and Schuell BioScience GmbH, Dassel, Germany). The detection of specific proteins was accomplished using antibodies, with β-actin serving as a loading control. Immunoactive proteins were visualized using an enhanced chemiluminescence Western blotting detection kit (Amersham Biosciences, Piscataway, NJ, USA). Protein levels were quantified using densitometry software (ImageLab, version 6.1.0, Bio-Rad, Hercules, CA, USA).

### 2.13. Statistics

The data are presented as the mean ± standard deviation (SD). Statistical analysis was conducted using the GraphPad Prism 9 software (GraphPad Software, Inc., San Diego, CA, USA). This study employed a one-way analysis of variance (ANOVA) followed by Dunnett’s post hoc test. *p* < 0.05 was considered significant.

## 3. Results

Scheme 1 illustrates simplified structures of the studied conjugates: AgNPs-CHL, in which chlorhexidine is attached to the surface via weak silver–nitrogen bonds, and AgNPs-MET-PEG, with metronidazol connected with the nanoparticles’ surfaces by a PEG linker via silver–sulfur bonds. Importantly, regarding chlorhexidine’s structure, theoretically, one molecule of this drug may contribute to stabilizing a few AgNPs, while the PEG linker in the AgNPs-MET-PEG is attached to the silver surface via two Ag-S bonds.

### 3.1. UV–Vis Studies of Stability of Nanoparticles in Cell Culture Media Environment

**Figure 1:**
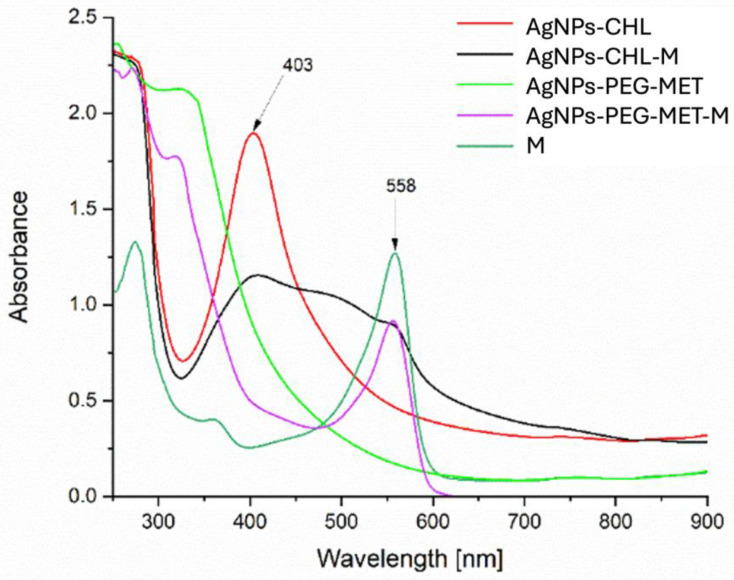
UV–Vis spectra recorded for the aqueous solution of nanoparticles and for these solutions with the addition of the cell culture medium (labeled with an M) and an aqueous solution of the medium (labeled as M).

Figure 1 displays the UV–Vis spectra recorded for the aqueous solutions of the studied nanoparticles and with the addition of the cell culture medium. As is visible in Figure 1, after adding the medium for cell culturing (denoted as M), the spectrum for the AgNPs-PEG-MET is practically unchanged, with one noticeable change—namely, the band’s presence, with a maximum at 558 nm from the medium. Contrary to AgNPs-CHL, the surface plasmon resonance band (SPR), with a maximum of 403 nm after adding the medium, is shifted to a longer wavelength and significantly broadened.

**Figure 2:**
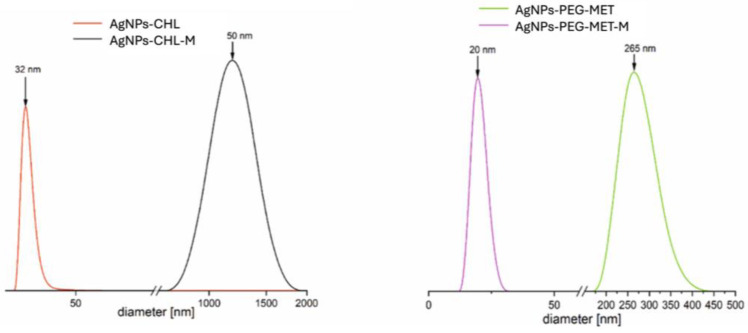
The hydrodynamic diameter distributions (calculated as the number average) of the AgNPs were determined by DLS measurements in aqueous solutions without and with the addition of the medium used for the cultivation of cells (the curves labeled as M).

Figure 2 shows the distribution of the hydrodynamic diameters determined in aqueous solutions and the medium used for cell culturing (labeled with an M). In the case of AgNPs-CHL in a water solution, the average hydrodynamic diameter of the nanoparticles is approximately 30 nm, and, after the medium’s addition, it increases to above 1000 nm. Contrary to the changes observed for AgNPs-CHL, the hydrodynamic diameter decreases from 200 nm to 20 nm after the medium’s addition for AgNPs-PEG-MET.

Table 1 displays the results of the ς-potential measurements in the same solutions taken for the DLS studies. The AgNPs-CHL had positive surface charges with high values, which decreased after adding the culturing media but remained positive. In contrast, the surface charge of the AgNPs-PEG-MET was negative and increased after adding the media.

**Figure 3:**
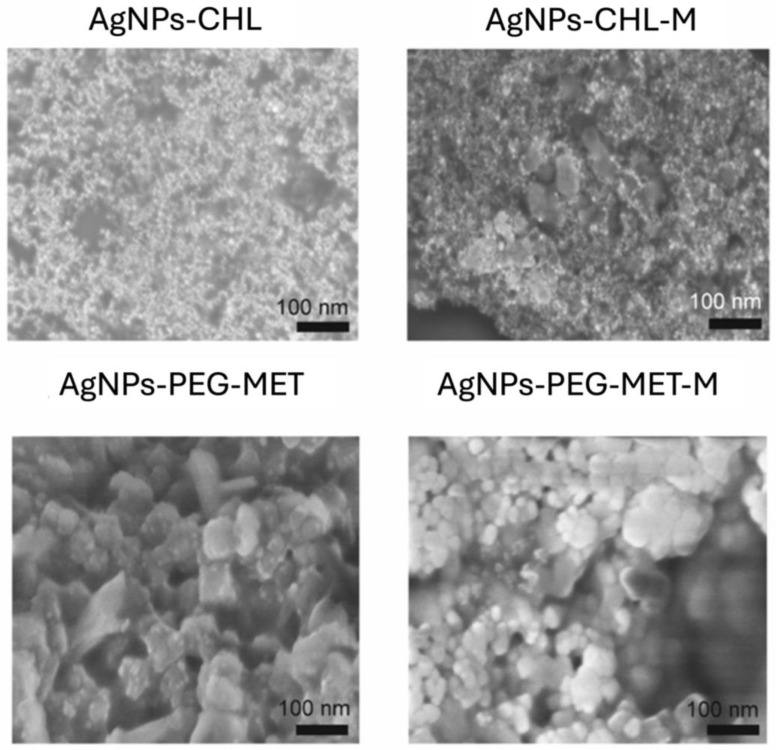
SEM images of the studied nanoparticles in solid form after water evaporation from their aqueous solutions, without and with the addition of the culture medium (denoted by M).

**Figure 4:**
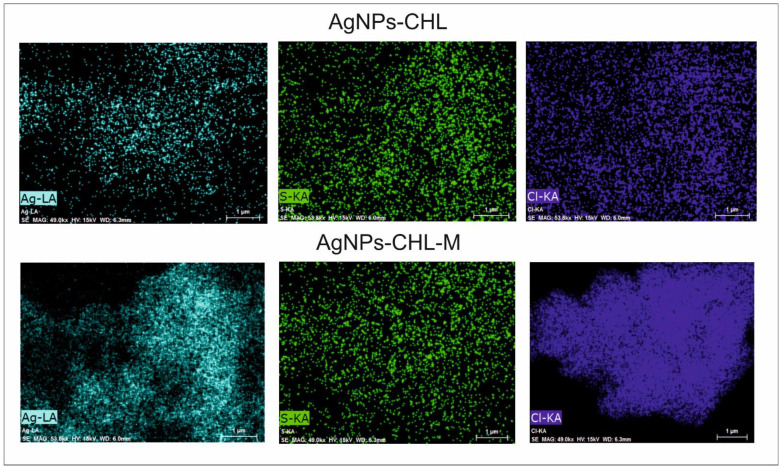
The maps of selected elements in the solid samples of AgNPs-CHL after water evaporation from their aqueous solutions and solutions containing the culture medium (denoted by M). The abbreviations LA and KA correspond to the L-alpha and K-alpha lines, respectively.

We also analyzed the changes in the nanoparticles’ morphologies after adding the culture medium in a solid state. Figure 3 displays the SEM images of the samples prepared from the solution of AgNPs without and with the addition of the medium as a result of water evaporation. The solutions were taken with the same concentrations as in the UV–Vis and DLS studies. EDS mapping was performed for the prepared samples to analyze the elemental composition, giving information about possible nanoparticle aggregation. Figure 4 shows maps of selected elements in the AgNPs-CHL samples recorded by SEM-EDS. Analogical maps for AgNPs-PEG-MET are presented in the Supplementary Information (Figure S1). The changes in the morphologies of the nanoparticles under the influence of environmental switching are also clearly visible in the results of the SEM-EDS studies. In this case, the differences after solvent removal can be observed. After the evaporation of water from the AgNPs-CHL solution, the nanoparticles are homogeneously dispersed, and their sizes are very similar throughout the whole sample (Figure 3). However, after solvent evaporation from the solution with the culture medium, agglomerates/aggregates with larger sizes are visible in the sample. This is more clearly visible when we analyze the maps of elements from the EDS measurements (Figure 4). Significant differences in the nanoparticles’ morphologies under the influence of environmental switching are also visible for the AgNPs-PEG-MET. As seen in Figure 3, small nanoparticles are located inside the nanocrystals formed by PEG-MET molecules. When the water solution is removed from the system with the culture medium, the formation of such crystals is disturbed, and irregular and smaller structures are created. Nevertheless, we do not observe significant differences in the element mapping from SEM-EDS between AgNPs-PEG-MET and the samples obtained in the presence of the culture medium (Figure S1). This indicates that, in this case, the aggregation of the nanoparticles does not occur under the influence of molecules in the culture medium.

### 3.2. Cytotoxicity Assessment

**Figure 5:**
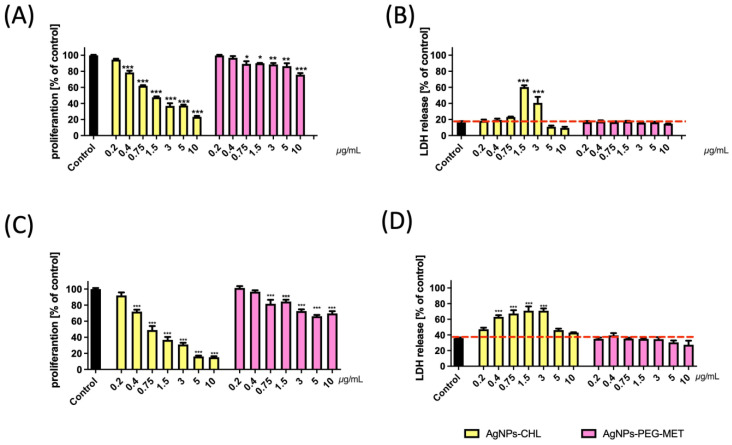
Cytotoxicity of AgNPs-CHL and AgNPs-PEG-MET after 24 h of incubation. Impact of nanoparticles on proliferation of (**A**) HGF-1 and (**C**) hFOB1.19 cells. Impact of NPs on LDH release from (**B**) HGF-1 and (**D**) hFOB1.19 cells. Data are presented as mean ± SD. * *p* < 0.05, ** *p* < 0.01, *** *p* < 0.001.

The AgNPs-CHL decreased cell proliferation at concentrations of 0.4 μg/mL and higher, whereas the AgNPs-PEG-MET were cytotoxic at concentrations of 0.75 μg/mL and higher. The cytotoxicity of the NPs was concentration-dependent. hFOB.19 cells were more susceptible to the NPs’ cytotoxic activity. Only AgNPs-CHL in the mid-concentration range caused increased LDH release from both cell lines (Figure 5).

### 3.3. Oxidative Stress Markers

**Figure 6:**
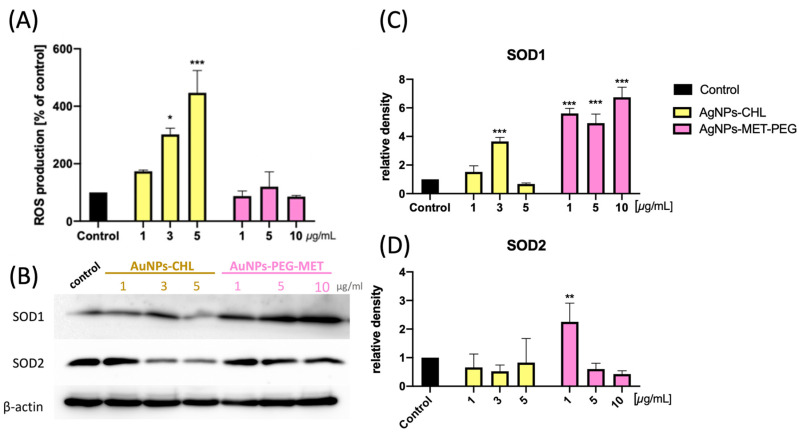
Impacts of AgNPs-CHL and AgNPs-PEG-MET on oxidative stress in HGF-1 cells. (**A**) Intracellular ROS production; (**B**) Western blot analysis of SOD1 and SOD2 production (Original images can be found in the Supplementary Materials.); densitometric analysis of (**C**) SOD1 and (**D**) SOD2. Data are presented as mean ± SD from *n* = 3 independent experiments. Statistical significance was determined using one-way ANOVA with Dunnett’s post hoc test. * *p* < 0.05, ** *p* < 0.01, *** *p* < 0.001.

Only AgNPs-CHL caused a significant increase in intracellular ROS production. However, both AgNPs-CHL and AgNPs-PEG-MET caused an imbalance in the expression of SOD proteins (Figure 6).

### 3.4. Cell Cycle Distribution

**Figure 7:**
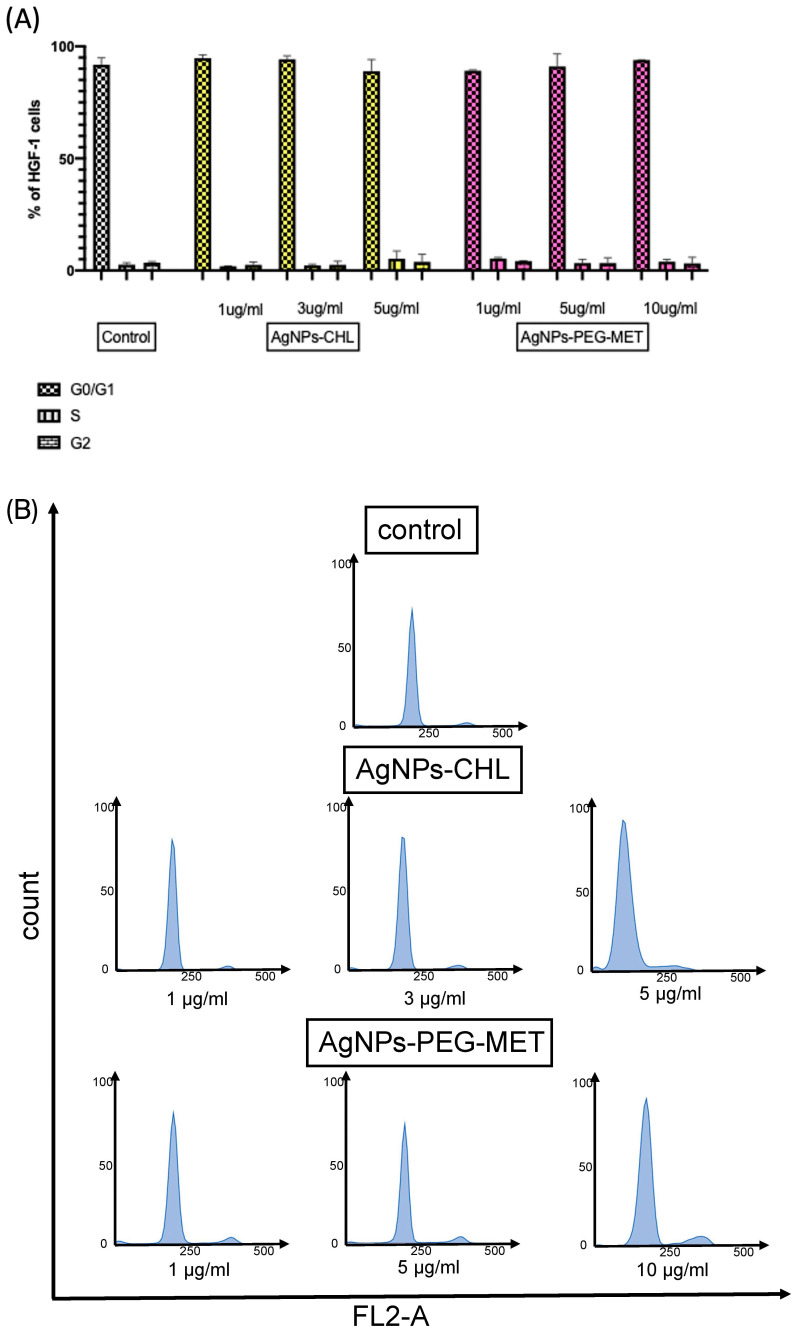
The cell cycle distribution for HGF1 cells. (**A**) Percentage of cells in each cell cycle phase after treatment, with (**B**) representative histograms. Data are presented as mean ± SD.

The AgNPs-CHL and AgNPs-PEG-MET did not impact the cell cycle distribution (Figure 7).

### 3.5. Cell Death Induction

**Figure 8:**
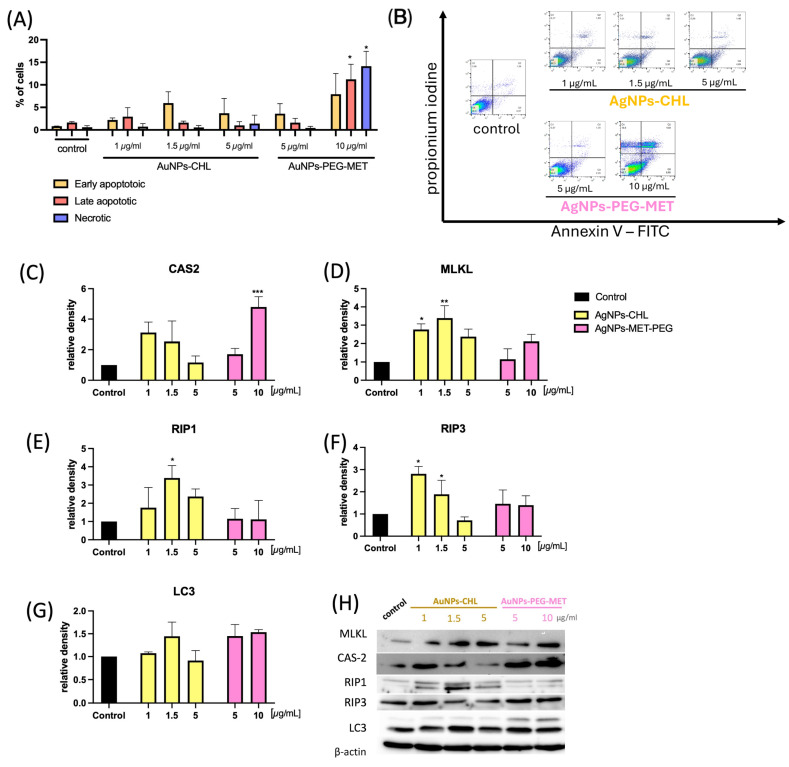
Analysis of apoptosis of HGF-1 cells after 24 h of incubation with AgNPs-CHL or AgNPs-PEG-MET. (**A**) Percentage of cells in early/late apoptotic or necrotic state. (**B**) Representative bivariate histograms and densitometric analysis of (**C**) CAS2, (**D**) MLKL, (**E**) RIP1, (**F**) RIP3, and (**G**) LC3. (**H**) Representative Western blot films. Data are presented as mean ± SD from *n* = 3 independent experiments. Statistical significance was determined using one-way ANOVA with Dunnett’s post hoc test. * *p* < 0.05, ** *p* < 0.01, *** *p* < 0.001. Original images can be found in the Supplementary Materials.

AgNPs-CHL did not cause an increased number of apoptotic cells. However, if the cells were incubated with AgNPs-CHL, increased protein levels of MLKL, RIP1, and RIP3 were observed. A higher concentration of AgNPs-PEG-MET caused increased apoptotic cells. Moreover, incubation with AgNPs-PEG-MET caused the concentration-dependent increased production of caspese-2 (CAS2) (Figure 8).

## 4. Discussion

We determined that AgNPs-CHL and AgNPs-PEG-MET decreased the cell proliferation rate; however, only AgNPs-CHL caused significantly increased LDH release. Both NP types caused changes in antioxidant protein production. However, only AgNPs-CHL were associated with increased intracellular ROS production. AgNPs-CHL caused increased levels of necroptosis-related proteins (RIP1, RIP3, MLKL). AgNPs-PEG-MET caused apoptotic cell death, which was confirmed by flow cytometry and increased CAS2 production. To summarize, AgNPs-CHL caused necroptosis and AgNPs-PEG-MET caused apoptosis.

Recent studies further support the therapeutic relevance of silver nanoparticle formulations conjugated with chlorhexidine or metronidazole in the treatment of periodontitis. Savkina et al. demonstrated that metronidazole-loaded silver nanoparticles embedded in alginate gel significantly improved periodontal tissue repair in a rat model [21]. Moreover, Wong et al. developed a silver nanoparticle-based antimicrobial hydrogel that effectively targeted key periodontal pathogens and showed promise for imporved tissue regeneration [22]. Similarly, Fahmy reported that combining silver nanoparticles with chlorhexidine enhanced dentin’s microhardness and exhibited superior antimicrobial properties compared to either agent alone, reinforcing the value of dual-function nanosystems in dental applications [23]. The growing clinical interest in AgNP-based hybrid materials aligns with our findings on the differential cytotoxic and functional profiles of AgNPs-CHL and AgNPs-PEG-MET.

The UV–Vis spectra gave possible reasons for the differences in the toxicity of AgNPs-CHL and AgNPs-PEG-MET. The observed changes in the surface plasmon resonance for AgNPs-CHL indicate that the nanoparticles grow under the influence of environmental changes (the dielectric constant is significantly higher after culture medium addition and in the presence of ionic species), and their polydispersity increases [24]. The SPR band is combined with the band from the medium in one broad band. The observed differences are caused by the different means of connecting the ligands on the nanoparticles’ surfaces. Chlorhexidine is attached to the surface via weaker bonds, creating bonds between nitrogen atoms in its molecule and the silver surface; meanwhile, metronidazole is connected with a PEG molecule, and this molecule is attached via a stronger, covalent Ag-S bond. Therefore, after the addition of the cell medium, chlorhexidine could be replaced by molecules from the medium that do not protect the silver surface against aggregation, leading to the formation of larger particles with an exposed silver surface from which silver ions are easily released (increasing the toxicity), and, on this surface, ROS can be formed. The results of the DLS measurements are consistent with the insights from the UV–Vis studies. Figure 2 shows how the hydrodynamic diameters of the AgNPs change after adding the medium. Notably, the hydrodynamic size includes a metal core, stabilizing layer, and hydration sphere [25]. We observed a drastic increase in the average hydrodynamic diameter of AgNPs-CHL. The aggregation process of the nanoparticles can explain such changes as the chlorhexidine layer is destabilized by the molecules from the medium.

Interesting results were obtained in these studies for AgNPs-PEG-MET. Contrary to the changes observed for AgNPs-CHL, the hydrodynamic radius decreased after the medium’s addition. This could be related to changes in the solvation layer after the addition of the medium containing many hydrophilic compounds, such as proteins, amino acids, salts, and saccharides, which have very high affinity to water molecules; thus, the hydration spheres on the AgNPs-PEG-MET shrink, and the hydrodynamic diameter decreases. This phenomenon can be explained as the shrinking of macromolecular coils formed by PEG molecules under the influence of salt solutions, which was observed for non-attached molecules of this polymer [26].

The changes in the structures of the nanoparticles after the addition of the culture medium are also visible in their averaged surface charges. Table 1 shows the results of the ζ-potential measurements for aqueous solutions of the studied AgNPs. For AgNPs-CHL, the surface charge in the water solution is positive, and its value is very high (57.4 mV). This indicates that the nitrogen atoms in chlorhexidine molecules attached to silver cores are mostly protonated. Thus, strong electrostatic interactions between these nanoparticles and the negatively charged cell membranes are expected. Undoubtedly, this phenomenon enhances the penetration of the nanoparticles inside these cells and increases their toxicity. Adding a culture medium causes a decrease in the ζ-potential, but the total surface charge of the nanoparticles remains positive. For the AgNPs-PEG-MET, the total surface charge is negative, which can be explained by the deprotonated carboxylic groups in PEG molecules that are not connected with metronidazole (non-protonated carboxyl groups with negative charge). This indicates that the fraction on PEG linker molecules on the AgNP surface are not connected with metronidazole; thus, they can dissociate, and the averaged surface charge is negative. Due to the negative surface charge, the interactions of such nanoparticles with the membranes of living cells are weaker than for AgNPs-CHL, which is probably an important factor in determining the lower toxicity of AgNPs-PEG-MET compared to AgNPs-CHL. Notably, adding a medium to the solution of AgNPs-PEG-MET causes the surface charge to become less negative; thus, the interactions with living cells are more robust than in water. Most probably, ionized carboxyl groups are neutralized by cations from culture medium components (salts and amino acids). Furthermore, the observed shrinking of the PEG layer coating AgNPs in the culture medium may also explain the reduced toxicity of PEG-ylated silver nanoparticles compared with others. Such a form protects the silver surface better, and the production of ROS can be limited.

The changes in the morphologies of the nanoparticles under the influence of the environment are also clearly visible from the results of the SEM-EDS studies (Figure 3 and Figure 4). In the AgNPs-CHL, silver, sulfur, and chloride are evenly distributed, indicating that the silver nanoparticles are coated with epichlorohydrin and not aggregated after water evaporation. The maps for the AgNPs-CHL-M show the presence of clusters of silver and chloride atoms. Thus, it can be concluded that molecules from the medium solution destabilize the chlorhexidine layer, forming larger particles and probably a less coated silver surface. These insights are consistent with those obtained from the spectrophotometric and DLS studies.

There is well-established knowledge regarding AgNPs’ cytotoxicity. Carlson et al. reported that the impact of AgNPs on macrophage viability depends on their size and concentration. The smaller and more concentrated the AgNPs were, the more cytotoxic they were [10]. We also observed concentration-dependent cytotoxicity and that AgNPs conjugated with PEG were less cytotoxic. Interestingly, spherical nanoparticles have less cytotoxicity than “pointy” ones [27]. Moreover, the PEGylation of the nanoparticles decreased their cytotoxic potential [28]. One of the main mechanisms of AgNPs’ cytotoxicity is the generation of ROS. Smaller AgNPs are reported to be more potent in inducing ROS production [10]. ROS production may damage DNA and induce cell death [29]. Several studies have reported that AgNPs can impact the cell cycle. For example, cervical cancer cells (HeLa) underwent cell cycle arrest at the S and G2/M phases, resulting in an elevated subG1 population, after treatment with glucose-capped AgNPs [30]. A similar result was observed when prostatic cancer cells (DU145) were exposed to green synthesized silver nanoparticles [31]. Often, cell cycle arrest is ROS-mediated [32]. Contrary to these reports, we did not observe significant changes in cell cycle distribution, potentially due to the small impact of AgNPs-CHL and no impact of AgNPs-PEG-MET on ROS production. AgNPs exhibit no specific cell death mechanism. Reports suggest that AgNPs may cause apoptosis [33], necrosis [34], or autophagy [35]. Some reports even indicate that AgNPs may cause mixed-type cell death (apoptosis or necroptosis/necrosis, as well as autophagy and mitotic catastrophe) in pancreatic cancer cells (PANC-1) [36]. Interestingly, the functionalization of AgNPs may impact these processes. It was reported that citrate-capped AgNPs could induce apoptosis in HaCaT cells, whereas those functionalized with PEG did not [28]. Moreover, other studies have shown that, if citrate-coated AgNPs were functionalized with lactose or oligonucleotide, their potency to induce apoptosis decreased [37]. However, data on how capping impacts cell death caused by AgNPs are unfortunately limited.

Apoptosis is considered a regulated form of cell death, typically characterized by low potential to cause inflammation [38]. In contrast, necroptosis involves cellular rupture and the release of damage-associated molecular patterns (DAMPs), which may exacerbate inflammatory responses [39]. Thus, in periodontitis, where sustained inflammation drives tissue destruction, the induction of apoptosis, rather than necroptosis, may offer a clinically favorable safety profile. This suggests that the apoptotic response observed with AgNPs-PEG-MET will be determinant of higher biocompatibility in comparison to AgNPs-CHL. These findings are supported by prior studies demonstrating that nanoparticle formulations inducing apoptosis tend to show improved biocompatibility [40,41,42].

## 5. Conclusions

We determined the functionalization-dependent mechanism of cell death caused by silver nanoparticles with potential medical applications. An in-depth understanding of potential pharmaceuticals’ cytotoxicity is crucial for their safe usage. We also showed that the type of coating layer on the silver nanoparticles—especially the means of anchoring on the silver surface—significantly influences their toxicity due to its interactions with the living cells’ environment. Even the minor modification of AgNPs may substantially impact their properties, so it is essential to have an in-depth understanding of AgNPs’ cytotoxicity mechanisms to design more potent and safe therapeutics. DLS studies and potential measurements revealed that the structure of silver nanoparticles may be dramatically altered in a culture medium solution compared to what is observed in water. In the case of AgNPs covered with a loosely connected stabilizing layer (such as AgNPs-CHL), aggregation can be observed despite being stable in a water solution. This phenomenon was manifested by a dramatic increase in the size of the particles and can also provide exposure to silver surfaces, leading to the higher production of ROS. In the case of AgNPs coated with a PEG sphere, after the addition of a culture medium, a decrease in the hydration layer and significant changes in the total surface charge can be observed. The shrinking of the PEG layer around the silver core, under the influence of the culture medium, can provide tight coverage and reduce ROS production, resulting in the lower toxicity of these nanoparticles. A comparative assessment of the properties of both types of NPs is presented in Table 2.

## Figures and Tables

**Table 1:** The results of ς-potential measurements for aqueous solutions of nanoparticles without and with the addition of the culture medium (denoted with M) ^a^.

Solution	ς-Potential [mV]
AgNPs-CHL	57.4 ± 3.70
AgNPs-CHL-M	3.15 ± 0.05
AgNPs-PEG-MET	−14.2 ± 2.53
AgNPs-PEG-MET-M	−4.83 ± 1.25

^a^ The concentration of the nanoparticles was 500 ppm, with 200 µL of medium added per 1 mL of the nanoparticle solution.

**Table 2:** Comparative assessment of properties of AgNPs-CHL and AgNPs-PEG-MET.

Feature	AgNPs-CHL	AgNPs-PEG-MET
Aggregation	Strong aggregation in culture medium	Stable in medium, no significant aggregation
Surface Charge	Positive	Negative
ROS Production	Strong induction of ROS production	No significant ROS production
Cell Death Mechanism	Necroptosis	Apoptosis
Overall Cytotoxicity	Higher toxicity	Lower toxicity at equivalent concentrations

## Data Availability

The data that support the findings of this study are available within the article or from the corresponding author, upon reasonable request.

## Supplementary Materials

The following supporting information can be downloaded at: https://www.mdpi.com/article/10.3390/biom15060850/s1, Figure S1. The maps of selected elements in solid samples of AgNPs-PEG-MET after water evaporation from their aqueous solutions and solutions containing the culture medium (denoted by M). The abbreviations LA and KA correspond to L-alpha and K-alpha lines, respectively. All Western Blots’ original images can be found in the Supplementary Materials.
