# Supplementary material for: Functionalization-Dependent Cytotoxicity of Silver Nanoparticles: A Comparative Study of Chlorhexidine and Metronidazole Conjugates
Source: Biomolecules. 2025 Jun 10;15(6):850. doi: 10.3390/biom15060850 (PMC12191295; doi:10.3390/biom15060850)
Supplement: Supplementary file 1 [file biomolecules-15-00850-s001.zip › biomolecules-3610639-Figure S1.pdf]

biomolecules

MDPI

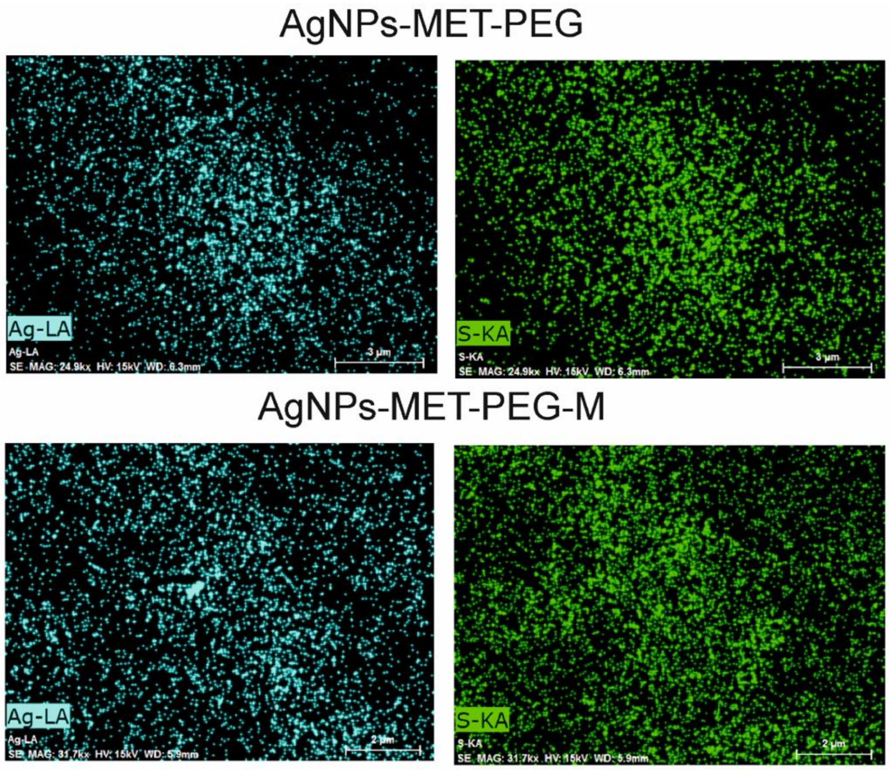

Figure S1. The maps of selected elements in the solid samples of AgNPs-MET-PEG after water evaporation from their aqueous solutions and solution containing the culturing medium (denoted with M letter). The abbreviations LA and KA correspond to L-alpha and K-alpha lines, respectively.
