# Supplementary material for: Functionalization-Dependent Cytotoxicity of Silver Nanoparticles: A Comparative Study of Chlorhexidine and Metronidazole Conjugates
Source: Biomolecules. 2025 Jun 10;15(6):850. doi: 10.3390/biom15060850 (PMC12191295; doi:10.3390/biom15060850)
Supplement: Supplementary file 1 [file biomolecules-15-00850-s001.zip › biomolecules-3610639-original images for Western Blot Experiment-Part 2.pdf]

Fig 6b, SOD1

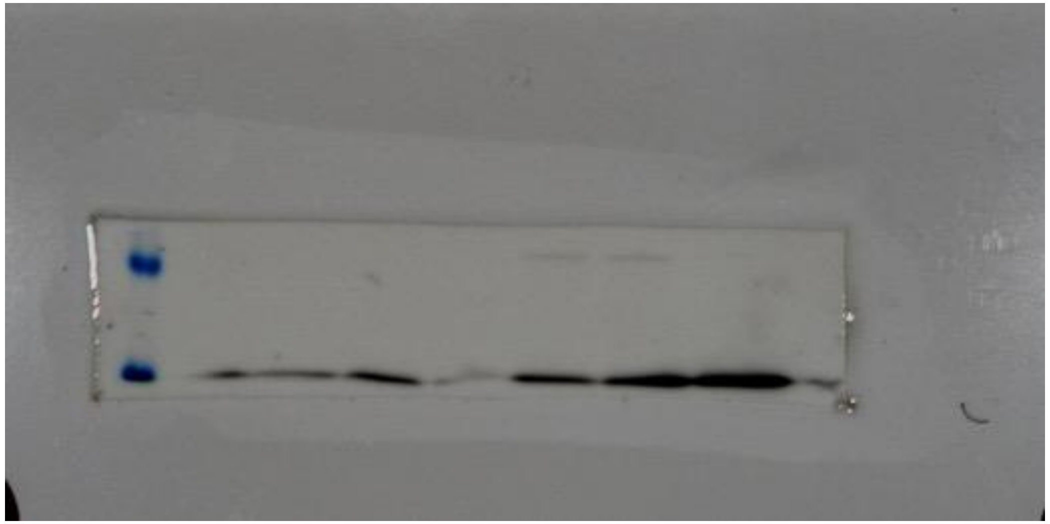

The loading order is consistent with that shown in the figure within the manuscript body.

Fig 6b, SOD2

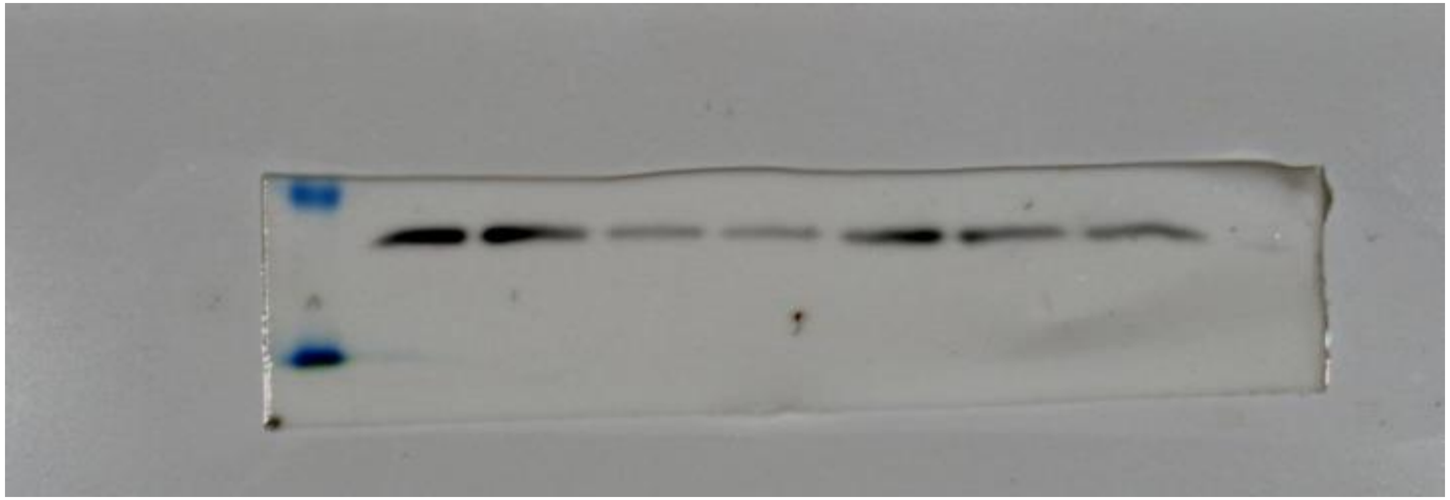

The loading order is consistent with that shown in the figure within the manuscript body.

Fig 6b, β-Actin

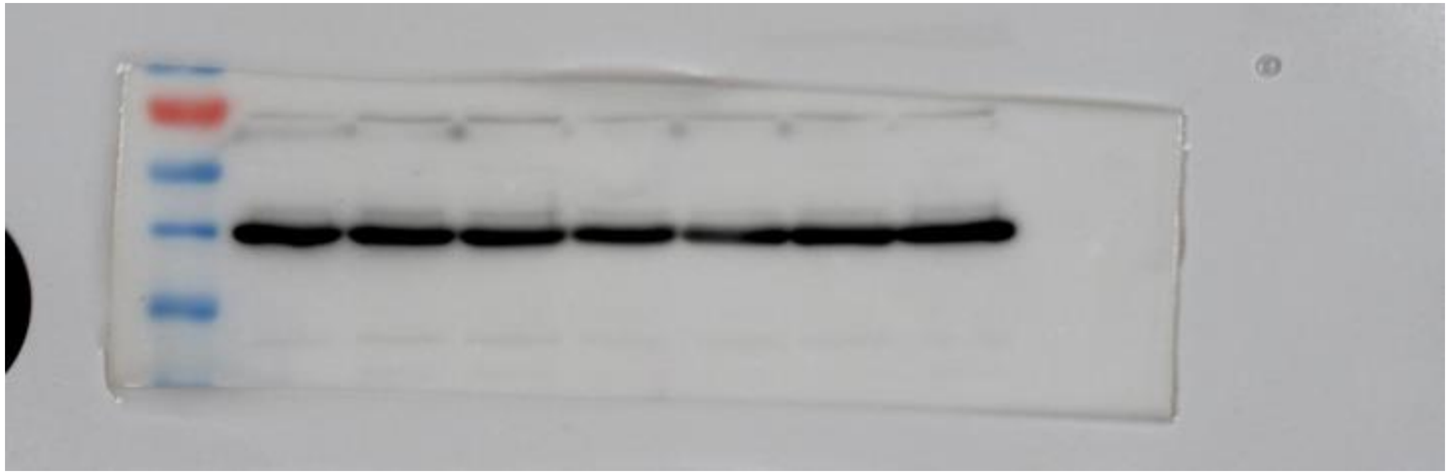

The loading order is consistent with that shown in the figure within the manuscript body.

Fig 8h, MLKL

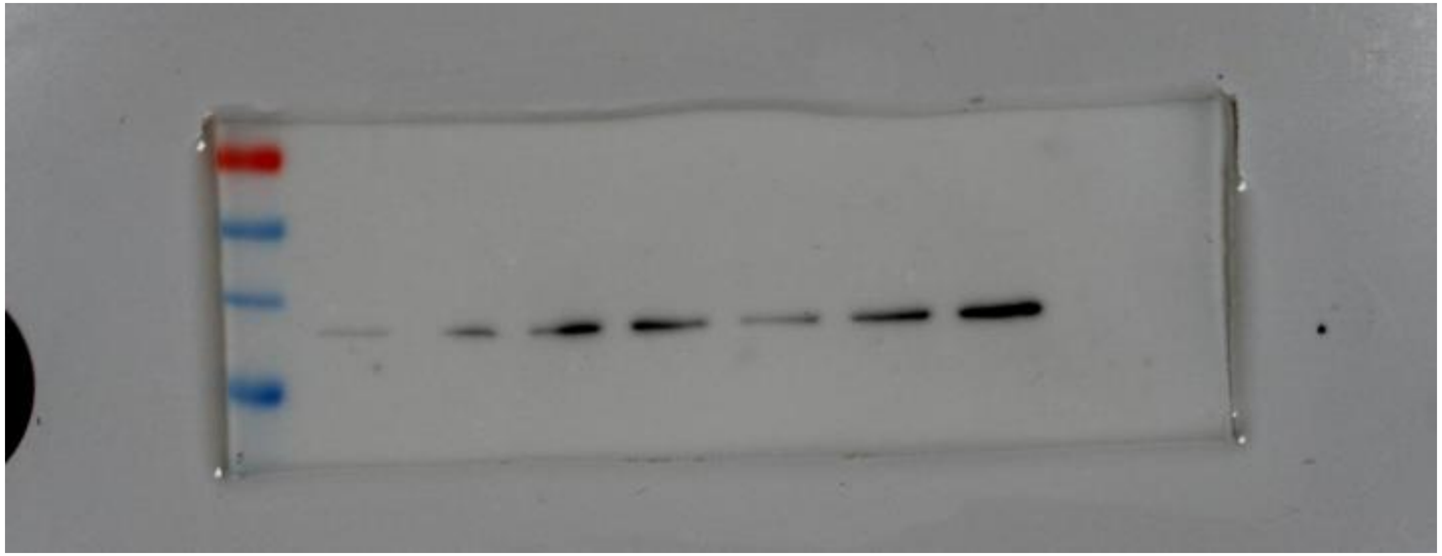

Loading order is the same as in the figure within the manuscript body. The last lane represents concentration of AgNPs-PEG-MET not presented in the paper.

Fig 8h, CAS2

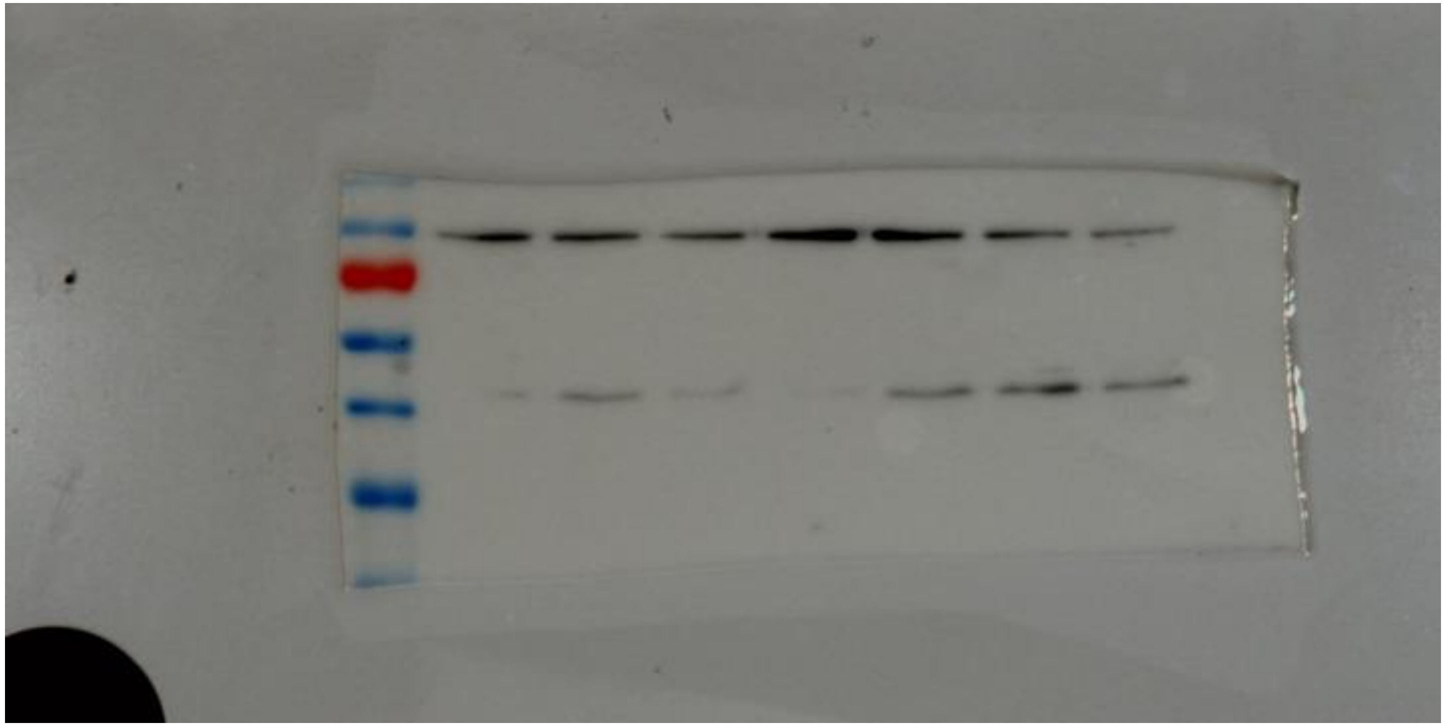

The loading order is consistent with that shown in the figure within the manuscript body. The last lane corresponds to a concentration of AgNPs-PEG-MET that is not presented in the paper.

Fig 8h, RIP1

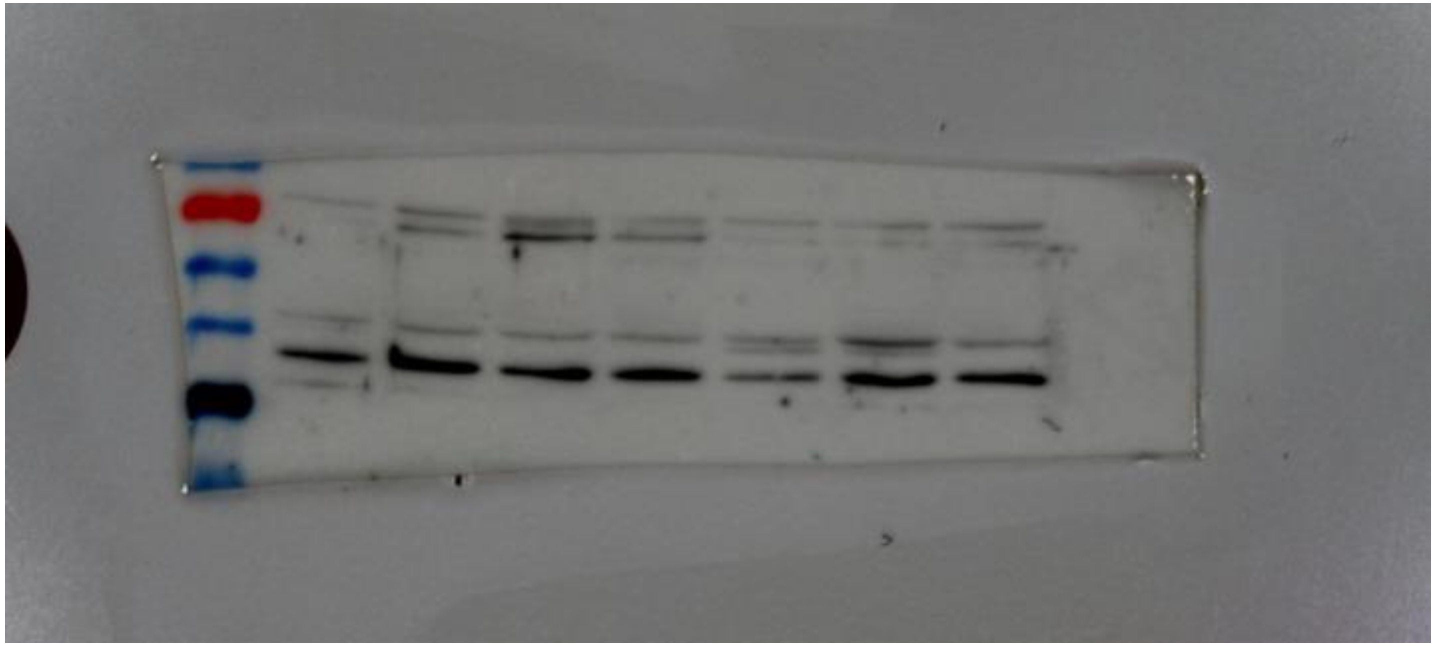

The loading order is consistent with that shown in the figure within the manuscript body. The last lane corresponds to a concentration of AgNPs-PEG-MET that is not presented in the paper.

Fig 8h, RIP3

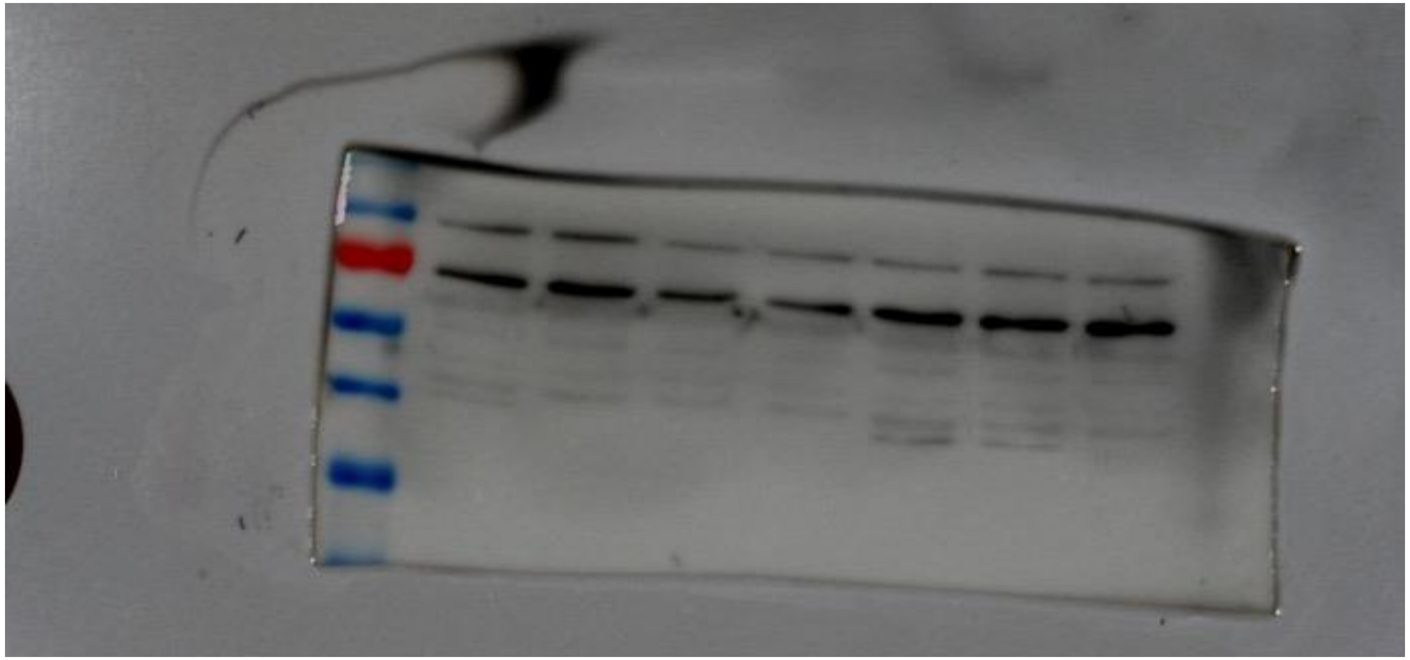

The loading order is consistent with that shown in the figure within the manuscript body. The last lane corresponds to a concentration of AgNPs-PEG-MET that is not presented in the paper.

Fig 8h, LC3

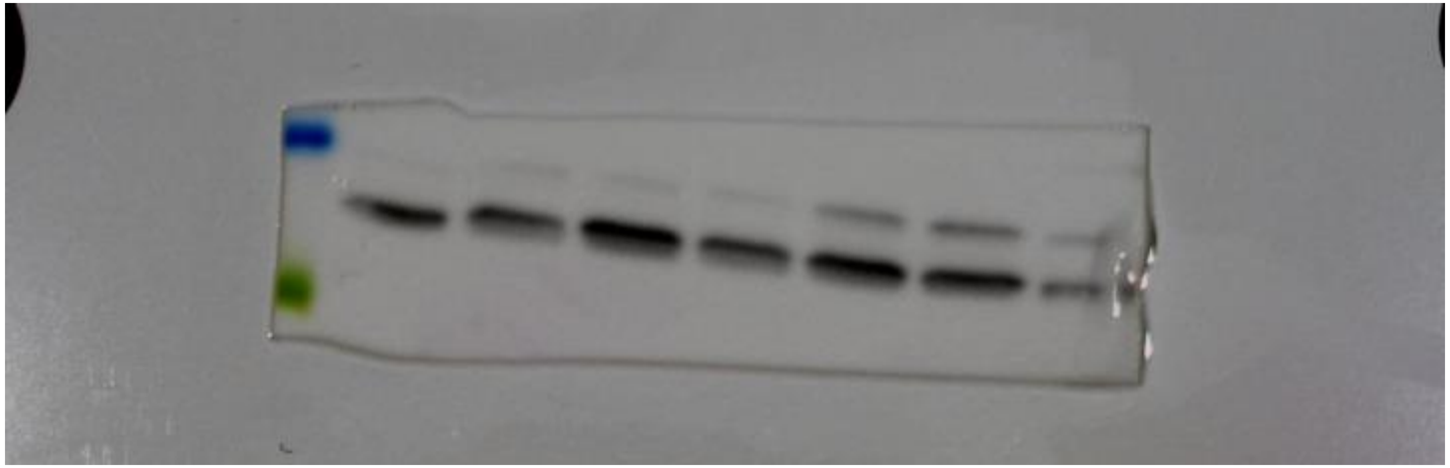

The loading order is consistent with that shown in the figure within the manuscript body. The last lane corresponds to a concentration of AgNPs-PEG-MET that is not presented in the paper.

Fig 8h, β-Actin

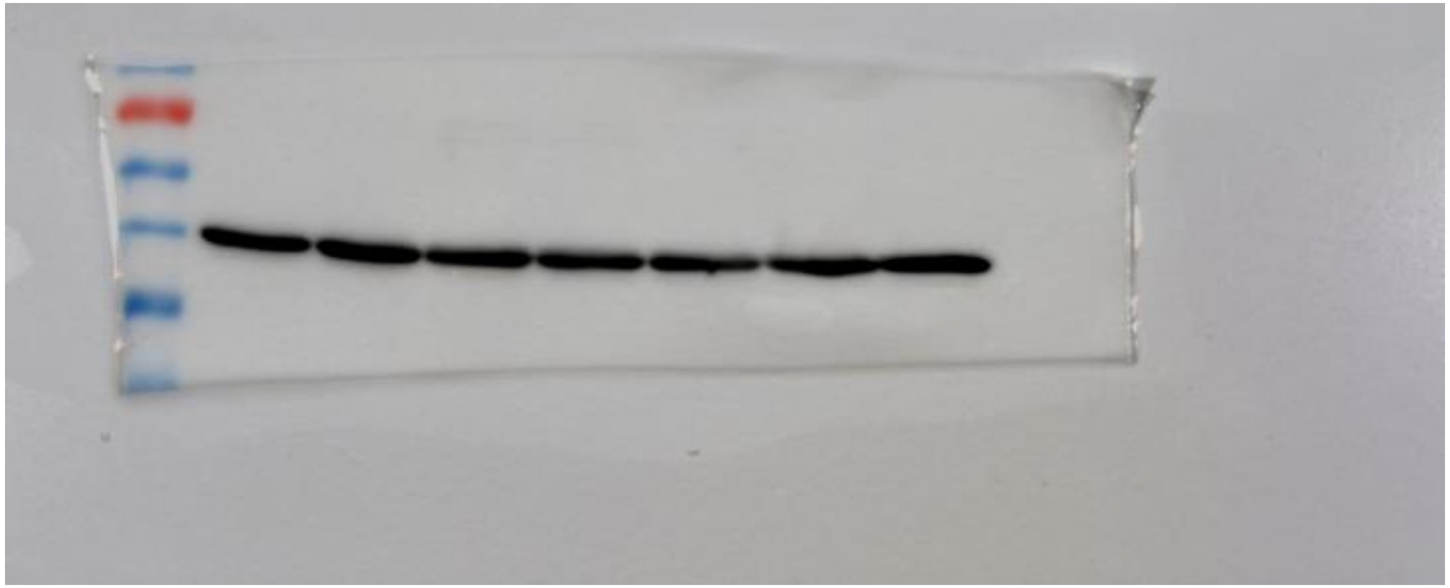

The loading order is consistent with that shown in the figure within the manuscript body. The last lane corresponds to a concentration of AgNPs-PEG-MET that is not presented in the paper.
